# Cytobiology of Human Prostate Cancer Cells and Its Clinical Applications

**DOI:** 10.3390/jcm8101716

**Published:** 2019-10-17

**Authors:** Kenichiro Ishii

**Affiliations:** Department of Oncologic Pathology, Mie University Graduate School of Medicine, Tsu, Mie 514-8507, Japan; kenishii@clin.medic.mie-u.ac.jp; Tel.: +81-59-232-1111

The number of males diagnosed with prostate cancer (PCa) is increasing all over the world [1]. Most patients with early-stage PCa can be treated by the appropriate therapy, such as radical prostatectomy or irradiation. On the other hand, androgen deprivation therapy (ADT) is the standard systemic therapy given to patients with advanced PCa. ADT induces temporary remission, but the majority of patients (approximately 60%) eventually progress to castration-resistant prostate cancer (CRPC), which is associated with a high mortality rate [2].

Generally, well-differentiated PCa cells are androgen-dependent, i.e., androgen receptor (AR) signaling regulates cell cycle and differentiation. Loss of AR signaling after ADT triggers androgen-independent outgrowth, generating poorly differentiated, uncontrollable PCa cells [3]. Once PCa cells lose their sensitivity to ADT, effective therapies are limited. In the last few years, however, several new options for the treatment of CRPC have been approved, e.g., the CYP17 inhibitor, the AR antagonist, and the taxane [4]. Despite this progress in the development of new drugs, there is a high medical need for optimizing the sequence and combination of approved drugs. Thus, identification of predictive biomarkers may help in the context of personalized medicine to guide treatment decisions, improve clinical outcomes, and prevent unnecessary side effects.

Departments of Nephro-Urologic Surgery and Andrology (Professor Emeritus Yoshiki Sugimura) and Oncologic Pathology (Professor Emeritus Taizo Shiraishi), Mie University Graduate School of Medicine, organized the semi-closed symposium on Biology of Prostate Gland Ise-Shima. This symposium was started in 2002 and was held every four years in 2006, 2010, 2014, and 2018 without any financial support from pharmaceutical companies and chemical industries. Each year, the symposium was attended by 40–50 Japanese investigators with expertise and interest in biology of the prostate gland and PCa. The goal of this symposium was to discuss the biological mechanism of the development and progression of prostatic proliferative diseases such as benign prostatic hyperplasia (BPH) and PCa. Several major topic areas were discussed, e.g., the pathophysiology of BPH, the tumor microenvironment of PCa, AR signaling in PCa progression, and the development of PCa detection and diagnosis. This Special Issue includes the major topics discussed at the symposium in 2018.

In this Special Issue, we focused on cytobiology of human PCa cells and its clinical applications to develop a major step towards personalized medicine matched to the individual needs of patients with early-stage and advanced PCa and CRPC. We hope that this Special Issue attracts a lot of attention for readers with expertise and interest in the cytobiology of human PCa cells and its clinical applications.
